# Large-scale plurimodal networks common to listening to, producing and reading word lists: an fMRI study combining task-induced activation and intrinsic connectivity in 144 right-handers

**DOI:** 10.1007/s00429-019-01951-4

**Published:** 2019-09-07

**Authors:** Isabelle Hesling, L. Labache, M. Joliot, N. Tzourio-Mazoyer

**Affiliations:** 1 University of Bordeaux, IMN, UMR 5293, 33000 Bordeaux, France; 2 CNRS, IMN, UMR 5293, 33000 Bordeaux, France; 3 CEA, GIN, IMN, UMR 5293, 33000 Bordeaux, France; 4 University of Bordeaux, IMB, UMR 5251, 33405 Talence, France; 5 INRIA Bordeaux Sud-Ouest, CQFD, INRIA, UMR 5251, 33405 Talence, France; 6 IMN Institut des Maladies Neurodégénératives UMR 5293, Team 5: GIN Groupe d’imagerie Neurofonctionnelle, CEA-CNRS, Université de Bordeaux, Centre Broca Nouvelle-Aquitaine-3ème étage, 146 rue Léo-Saignat-CS 61292-Case 28, 33076 Bordeaux CEDEX, France

**Keywords:** Word production, Word perception, Word reading, Hemispheric specialization, Resting state

## Abstract

We aimed at identifying plurimodal large-scale networks for producing, listening to and reading word lists based on the combined analyses of task-induced activation and resting-state intrinsic connectivity in 144 healthy right-handers. In the first step, we identified the regions in each hemisphere showing joint activation and joint asymmetry during the three tasks. In the left hemisphere, 14 homotopic regions of interest (hROIs) located in the left Rolandic sulcus, precentral gyrus, cingulate gyrus, cuneus and inferior supramarginal gyrus (SMG) met this criterion, and 7 hROIs located in the right hemisphere were located in the preSMA, medial superior frontal gyrus, precuneus and superior temporal sulcus (STS). In a second step, we calculated the BOLD temporal correlations across these 21 hROIs at rest and conducted a hierarchical clustering analysis to unravel their network organization. Two networks were identified, including the WORD-LIST_CORE network that aggregated 14 motor, premotor and phonemic areas in the left hemisphere plus the right STS that corresponded to the posterior human voice area (pHVA). The present results revealed that word-list processing is based on left articulatory and storage areas supporting the action–perception cycle common not only to production and listening but also to reading. The inclusion of the right pHVA acting as a prosodic integrative area highlights the importance of prosody in the three modalities and reveals an intertwining across hemispheres between prosodic (pHVA) and phonemic (left SMG) processing. These results are consistent with the motor theory of speech postulating that articulatory gestures are the central motor units on which word perception, production, and reading develop and act together.

## Introduction

Language is one of the most important and specific cognitive abilities of human beings. According to De Saussure (1975), language is a universal structure encompassing the abstract, systematic rules and conventions of a unifying system, which is independent of individual users, while speech is the personal use of language, thus presenting many different variations such as style, grammar, syntax, intonation, rhythm, and pronunciation. Though neuroimaging studies of language at the word/phonological level have demonstrated bilateral activation during language tasks, calculation of asymmetries provides results that are consistent with the neuropsychological evidence that language is implemented in large areas located along the left sylvian fissure (Vigneau et al. 2006, 2011). More specifically, word processing is underpinned by cortical areas involved in the auditory, visual, and motor areas spreading over the left hemisphere depending on the type of language modality (Price 2010, 2012). However, the question of the existence of core areas independent of the modality of the language task is still open. Regarding the left hemisphere arrangement of language areas, two divergent theories explain the relations of speech perception and speech production to language. The former, called the horizontal view, proposes that the elements of speech are sounds that rely on two separate processes (one for speech perception, the other for speech production) that are not specialized for language until a cognitive process connects them to each other and then to language (Fodor 1983). The latter, called the vertical view (or motor theory of speech perception), posits that speech elements are articulatory gestures serving both speech perception and production processes that are immediately linguistic, thus requiring no cognitive process (Liberman and Whalen 2000). More generally, the existence of a bilateral dorsal–ventral model of speech processing with preferential leftward involvement has been widely accepted (Binder et al. 1996; Hickok and Poeppel 2004; Rauschecker and Tian 2000). This model posits the coexistence of (1) a dorsal pathway, i.e. the “where stream,” in which an acoustic–phonetic–articulatory transformation linking auditory representations to motor representations is reported to occur in superior temporal/parietal areas and ultimately in frontal areas (Buchsbaum et al. 2001); and (2) a ventral pathway, i.e. the “what stream”, in which speech-derived representations interface with lexical semantic representations, reported to involve the superior, middle, and inferior temporal gyri (Binder et al. 2000; Hickok and Poeppel 2000). Interestingly, concerning the dorsal pathway, the postulated existence of an auditory–motor system (Hickok and Poeppel 2000) has been supported by studies that aimed at examining the role of motor areas in speech perception. Hence, an fMRI study revealed that listening to syllables and producing the same syllables led to a common bilateral network encompassing a superior part of the ventral premotor cortex, suggesting the existence of a common phonetic code between speech perception and production (Wilson et al. 2004). Furthermore, another study has not only suggested that the cortical motor system is organized in a somatotopic way along the precentral cortex with the lip area being superior to the tongue area, but also revealed that these precentral regions are consistently activated by syllable articulation and syllable perception, hence demonstrating a shared speech-sound-specific neural substrate of these sensory and motor processes (Pulvermüller et al. 2006). These findings were supported by a meta-analysis revealing that in right-handers, activations of the posterior part of the frontal lobe distributed along the precentral gyrus were strongly left lateralized during both production and auditory tasks at the word or syllable level, together with the involvement of the supramarginal gyrus (SMG) (Vigneau et al. 2006). Moreover, a recent MEG study reported a synchronization between the anterior motor regions involved in syllable articulation and the posterior regions involved in their auditory perception during perception of these syllables (Assaneo and Poeppel 2018). In addition, studies on split-brain patients have demonstrated a strict leftward lateralization concerning phonological processing, with split-brain patients’ right hemisphere lacking categorical perception of phonemes (Gazzaniga 2000; Sidtis et al. 1981). Such a leftward lateralization was confirmed by studies using the Wada test procedure (Dym et al. 2011), and the leftward asymmetry of the audio–motor loop measured with functional imaging actually supports the left hemisphere specialization for the phonological processing of speech (Vigneau et al. 2006, Zago et al. 2008).

Though mastered afterwards, human beings have developed other ways of using language through other sensory modalities, such as the visual system in the case of reading. Accurate perception and production of speech sounds are essential for learning the relationship between sounds and letters. Phonological awareness, i.e. the ability to detect and manipulate speech sounds, or phonemes, is the best predictor of reading ability. Reading is based on both the ability to hear and segment words into phonemes and then to associate these phonemes with graphemes, with the mapping of orthographic to phonological representations during reading being intrinsically cross-modal (McNorgan et al. 2014). Research has revealed that a phonological processing deficit underlies reading difficulties in dyslexic children, establishing a link between perception and reading abilities (Gillon 2004). In the case of disorders of oral language development, specific language impairment (SLI) is the most frequently studied developmental disorder. Children with specific language impairment have been reported to present impairments in phonological processing, whether in phonological awareness or in phonological memory, which is evidence of a link between production and reading abilities; the neural support of this link still needs to be clarified (Catts et al. 2005). Different studies examining the word processing cerebral networks common to the auditory and visual modalities have revealed the supramodal involvement of anterior regions [supplementary motor area (SMA) and prefrontal, premotor and inferior frontal gyri], whereas variations have been observed in the temporal lobe depending on the language task (Booth et al. 2002a, b; Buckner et al. 2000; Chee et al. 1999), making it difficult to conclude the existence of a common antero-posterior network for plurimodal word processing. Regarding semantic processing, it should be noted that one study addressing production and reading in four languages revealed a common bilateral network involved in these two tasks (Rueckl et al. 2015). Moreover, since the complete development of speech in literate individuals leads to the mastering of the written language, we expected that the core word areas developing conjointly in the three modalities would include some visual areas, which would be part of a large-scale plurimodal network underpinning word processing. It is worth emphasizing that, even if less investigated, the first phase of speech acquisition in newborn babies is perceptual, as the infant hears others’ vocalizations, highlighting the importance of prosody in speech processing. Speech prosody, i.e. the musical aspects of speech, is an early-developing component of speech, which could be compared to a musical stave upon which phonemes would be placed (Locke and Pearson 1990). This perceptual phase is crucial considering the inability to learn spoken language or even normally babble when infants are born deaf (Oller and MacNeilage 1983) or in the case of wild children (Curtiss 1977). In other words, children have to listen to the prosody of their mother tongue to be able to reproduce it. Lesional studies have revealed that the tonal prosodic brain areas are located in the right hemisphere along the STS, which includes the posterior human voice area (pHVA), highlighting the potential role of these right hemispheric regions during development. The second phase of speech acquisition is production. In fact, children master the prosodic dimension before producing their first words (Bever et al. 1971). Production develops through the process of imitation, highlighting that prosodic processing is one element of the construction of a strong dependency between perception and production throughout development. This is illustrated, for example, by persisting difficulties in speech production encountered by infants who were tracheotomized at a time when they should have normally babbled (Locke and Pearson 1990). Interestingly, metre in speech, whose acoustic correlate is stress, has been revealed to be important for both speech perception (Jusczyk et al. 1993) and production (Gerken et al. 1990). Once the metrical rules (which provide important cues for speech segmentation within the continuous speech stream) have been acquired, a speech metre contributes to phonological (Pitt and Samuel 1990), semantic (Schwartze et al. 2011) and syntactic (Roncaglia-Denissen et al. 2013) processing. Musical rhythmic priming, using metres, has been revealed to enhance phonological production in hearing-impaired children due to an enhanced perception of sentences (Cason et al. 2015). Furthermore, in the context of speech rehabilitation therapies, musical rhythm has been revealed to be a fluency-enhancing tool (Thaut 2013). More generally, the prosodic dimension of speech has been used to restore the speech of Broca’s aphasic patients, and the term Melodic Intonation Therapy was coined to refer to this technique based on the use of melody and singing, which would be core musical elements predominantly engaging the right hemisphere (Thaut and McIntosh 2014). The right STS specialization for tonal processing was evidenced by a neuroimaging study as a rightward asymmetry of activation (Zatorre and Belin 2001). Other studies have highlighted the role of the right hemisphere, particularly the right STS, in the prosodic dimension of speech (Beaucousin et al. 2007; Belin et al. 2004; Sammler et al. 2015). Given the importance of prosody in language development, we hypothesized that in addition to left hemisphere participation, right hemispheric regions hosting the tonal dimension of speech prosody may be involved in all three tasks, i.e. production, perception and reading tasks.

In summary, previous studies on phonological/word processing have, at best, dealt with two different language modalities (either production and listening or production and reading) focusing on discrete cortical areas a priori selected (articulatory motor areas and SMG). In the present work, we assessed the three main language modalities: listening, production and reading. Furthermore, considering the importance of lateralization reported above, we took into consideration the right and left hemisphere contribution to task completion at the word or syllable levels in the present work. Finally, we integrated the connectivity data provided by a resting-state acquisition to propose a comprehensive view of the plurimodal large-scale networks for phonological/word processing and their potential roles.

To achieve the identification of plurimodal large-scale networks for word-list processing, a large population of 144 right-handers who completed word-list processing in the three modalities, production, reading and listening, during task-induced fMRI acquisition, was selected from the BIL&GIN database (Mazoyer et al. 2016). In this sample of healthy right-handers, we (1) identified left brain regions showing both leftward joint activation and leftward joint asymmetry and right brain regions showing both rightward joint activation and rightward joint asymmetry during the three word-list tasks; (2) identified the network organization at play within the areas previously identified based on the hierarchical clustering of the BOLD temporal correlation measured during a resting-state acquisition completed in the same individuals; and finally, (3) conducted a comprehensive investigation of how these areas were modulated according to the task and integrate the present results into the literature to elucidate the identified supramodal word-list network’s function/role.

## Materials and methods

### Participants

The present study included a sample of 144 right-handers balanced for sex (72 women) and picked from the BIL&GIN database, which is a multimodal imaging/psychometric/genetic database specifically designed for studying the structural and functional neural correlates of brain lateralization (Mazoyer et al. 2016). The selected participants had French as a mother tongue and were free from developmental disorders and neurological and psychiatric histories. A local ethics committee (CCPRB Basse-Normandie, France) approved the experimental protocol. The participants gave their informed, written consent and received an allowance for their participation. All subjects were free of brain abnormalities as assessed by an inspection of their structural T1-MRI scans by a trained radiologist.

The mean (± standard deviation) age of the sample was 27 ± 6 years (range 19–53), and the mean level of education (corresponding to the number of schooling years since the first grade of primary school) was 16 ± 2 years (range 11–20), corresponding to 4 years at the university level.

Handedness was self-reported by the subjects, and their manual lateralization strength was assessed using the Edinburgh inventory (Oldfield 1971) with values ranging from − 100 to + 100. The average MLS value of the subjects was 93.48 (SD = 11.49).

## Functional imaging

### Paradigm of the word-list tasks

Three runs were administered. The participants had to covertly generate (PROD), listen to (LISN) or covertly read (READ) lists of words (Word-List). These tasks were part of a run that alternated these word tasks with sentence tasks (see Labache et al. 2019 for the complete methodology).

**Fig. 1:**
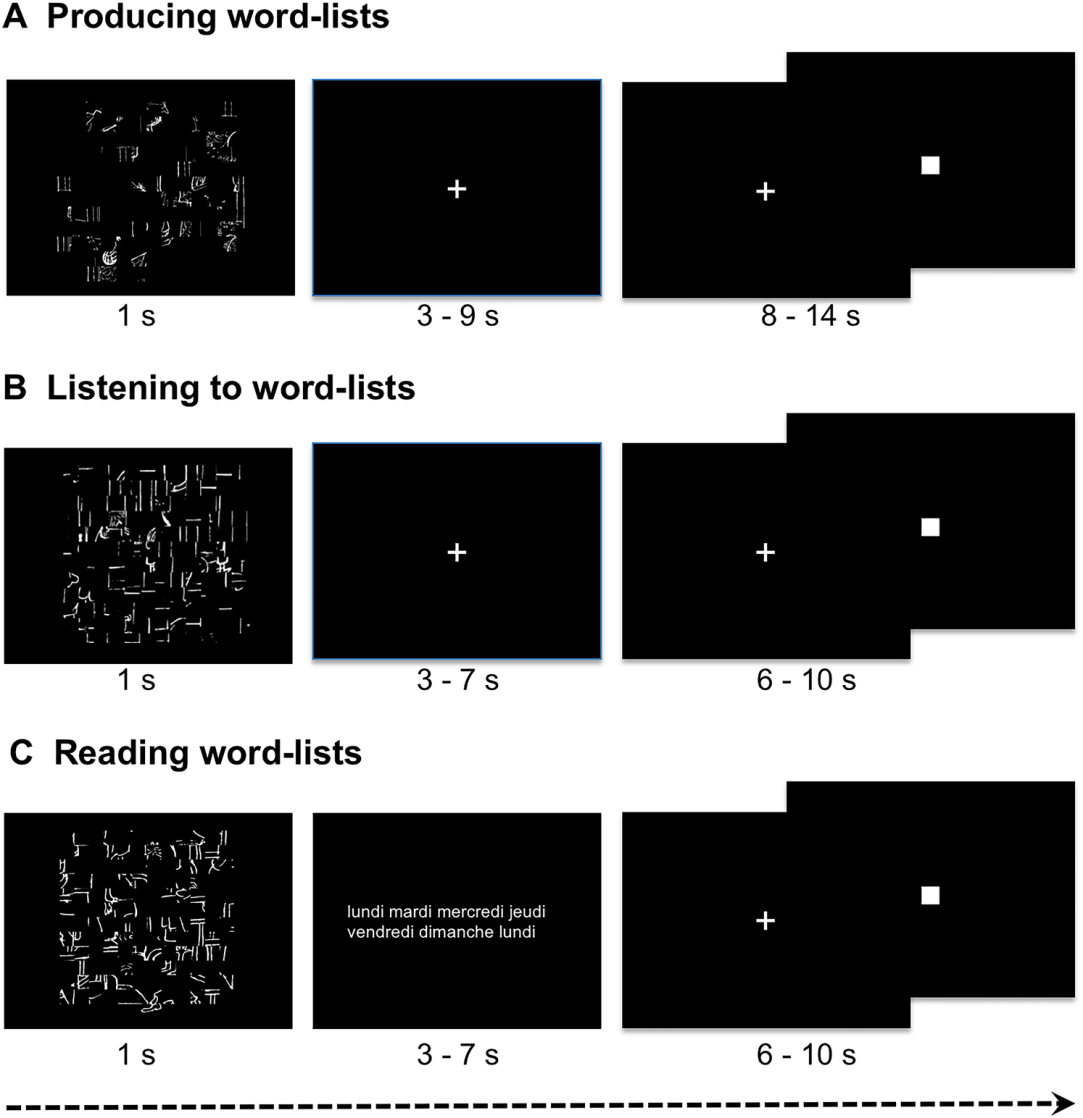
Description of the paradigm for one event from each Word-List task. In the three conditions, an event was initiated by the presentation of a scrambled picture for one second, followed by a central cross that participants were instructed to look at while they were producing the list of months of the year (**a**) or listening to a list of words (**b**). During the reading run, instead of a cross fixation, lists of words (either weeks, hours, seasons, days, months) were presented. The participants had to click when they had finished producing, listening to or reading, and they subsequently had to indicate by clicking when the central cross that reappeared was changed into a square. In this figure, for reading convenience, the stimuli were zoomed in on compared to the presentation of the stimuli during the scanning session

The stimuli were lists of over-learnt words, such as months of the year, making it possible to decrease the weight of lexico-semantic and syntactic processing and enhancing phonetic encoding, phonology, articulation and word retrieval while inducing prosodic processing due to the specific metrics of serial automatic speech. For each task, the participants were shown a scrambled drawing for 1 s, immediately followed by a central fixation crosshair (see Fig. 1). While fixating the cross, the subjects performed either the listening or production Word-List tasks and had to click on the response pad with their right hand after the task completion. During reading, the participants had to read lists of words that were flashed on the screen instead of fixating the cross, but still had to click on the response pad with their right hand after the task completion. Then, a low-level reference task followed each event (sentence or Word-List), consisting of sustaining visual fixation on the central cross and pressing the pad when the fixation cross was switched to a square (both stimuli covering a 0.8° × 0.8° visual area). This second part of the trial, which lasted at least half the total trial duration, aimed at refocusing the participant’s attention to a non-verbal stimulus and controlling for manual motor response activation, which was also present in the first part of the trial. This second part lasted from 8 to 14 s for the production task and from 6 to 10 s for the perception and reading tasks.

The design was almost identical for the three tasks. During PROD, the participants were asked to covertly generate the list of the months of the year from January to December during each of the ten Word-List trials, which lasted 18 s. During LISN, they were instructed to listen to 13 of 14-s trials of lists of the months, days of the week and/or seasons. During READ, they were asked to read lists of days of the weeks, months, and/or seasons that were flashed on the screen, and this task consisted of 13 trials lasting 14 s. Response times were recorded during the fMRI experiment for each event of each run for each participant. The mean response time corresponded to the mean response time of each of the 144 participants during a given task. Note that the participants responded with their right hand.

### Image acquisition and pre-processing

#### Structural imaging

Structural images were acquired using a 3T Philips Intera Achieva scanner and included high-resolution T1-weighted volumes [sequence parameters: repetition time (TR), 20 ms; echo time (TE), 4.6 ms; flip angle, 10°; inversion time, 800 ms; turbo field echo factor, 65; sense factor, 2; field of view, 256 × 256 × 180 mm^3^; isotropic voxel size, 1 × 1 × 1 mm^3^]. For each participant, the line between the anterior and posterior commissures was identified on a mid-sagittal section, and the T1-MRI volume was acquired after orienting the brain in the bi-commissural coordinate system. T2*-weighted multi-slice images were also acquired [T2*-weighted fast field echo (T2*-FFE); sequence parameters: TR = 3500 ms; TE = 35 ms; flip angle = 90°; sense factor = 2; 70 axial slices; isotropic voxel size, 2 × 2 × 2 mm^3^].

#### Task-induced image acquisition and analysis

The functional volumes were acquired as T2*-weighted echo-planar images (EPI) (TR = 2 s; TE = 35 ms; flip angle = 80°; 31 axial slices; field of view, 240 × 240 mm^2^; isotropic voxel size, 3.75 × 3.75 × 3.75 mm^3^). In the three runs, 192, 194 and 194 T2*-weighted volumes were acquired for the Word-List production, listening and reading tasks, respectively.

For each participant, the pre-processing of T2*-weighted echo-planar EPI images included (1) the T2*-FFE volume rigid registration to the T1-MRI; (2) the T1-MRI segmentation into three brain tissue classes: grey matter, white matter and cerebrospinal fluid; and (3) the T1-MRI scans normalization to the BIL&GIN template, which included 301 volunteers from the BIL&GIN database (aligned to the MNI space), using the SPM12 “normalize” procedure with otherwise default parameters. For each of the three fMRI runs, data were then corrected for slice timing differences and, to correct for subject motion during the runs, all the T2*-weighted volumes were realigned using a six-parameter rigid-body registration. The EPI-BOLD scans were then rigidly registered to the structural T2*-FFE image. The combination of all registration matrices allowed for warping the EPI-BOLD functional scans to the standard space with a single trilinear interpolation.

The initial analysis included the application of a 6-mm full-width at half-maximum Gaussian filter to each functional volume of each run. Global linear modelling [statistical parametric mapping (SPM), http://www.fil.ion.ucl.ac.uk/spm/] was then used to process the task-related fMRI data. For each participant, BOLD variations corresponding to each Word-List task versus the cross-change detection task belonging to the same run were computed [Word-List production (PROD), Word-List reading (READ), and Word-List listening (LISN)].

#### Homotopic regions of interest analysis

Since the brain presents a global torsion, the Yakovlevian torque, which prevents a perfect point-to-point correspondence between cortical areas that are functionally homotopic (Toga and Thompson 2003), the use of flipped images to calculate asymmetries appears problematic since the flipped regions do not correspond to the equivalent of the other hemisphere. A new atlas, called AICHA, based on resting-state fMRI data and composed of homotopic functional regions of interest (hROIs), has been devised to circumvent this problem and is thus suited for investigating brain hemispheric specialization and lateralization, allowing the determination of the right and left hemispheric contribution in language and for computing functional asymmetries in regions having equivalent intrinsic connectivity (Joliot et al. 2015).

BOLD signal variations were thus calculated for the right and left hROI BOLD signal variations for each of 185 pairs of functionally defined hROIs of the AICHA atlas (Joliot et al. 2015) adapted to SPM12 in the three contrast maps (defined at the voxel level) for PROD, LISN and READ.

## Part 1: identification and characterization of hROIs exhibiting both activation and asymmetry in all three tasks

To complete the identification of language areas underpinning production, listening and reading tasks at the word level, we first searched for hROIs that were both significantly co-activated and significantly co-asymmetrical on average among the 144 participants during the PROD, LISN and READ tasks for each hemisphere. The idea behind the conjunction of activations and asymmetries during the three tasks was to be selective enough to present brain areas specifically lateralized during the tasks. In a second step, we described the variation in activity and asymmetry in each hemisphere for the selected regions to obtain information on their functional nature from their modulation by the task component.

### Statistical analyses

#### hROIs selection

Using JMP14 (http://www.jmp.com, JMP® 2018), a conjunction analysis was conducted to select the left hemispheric hROIs exhibiting BOLD signal variations that were both significantly positive and significantly larger than those of their right counterparts in all three tasks. An hROI was selected whenever it was significantly activated in each of the three task contrasts using a significance threshold set to *p* < 0.05 per contrast. The significance threshold for the conjunction of activation in the three tasks was thus 0.05 × 0. 05 × 0.05 = 1.25 × 10^−4^. The second criterion for hROI selection was the existence of a significant leftward asymmetry in each of the three task contrasts, with the threshold of significance of this second conjunction being 1.25 × 10^−4^. Finally, to be selected, a given hROI had to fulfil both criteria, and the overall significance threshold for the conjunction of the conjunction analyses was 1.56 × 10^−8^ = (1.25 × 10^−4^)^2^. This procedure was conducted separately for the left and right hemispheres.

#### Characterization of hROI activations and asymmetries across tasks

Taking the right and left hROIs separately, we tested the existence of significant effects of Task (PROD, LISN, READ) on the selected hROIs, as well as the effect of Side (left or right) and their interactions, using two repeated-measures linear mixed-effects models. The analysis was conducted on the 144 individuals entering the variable Subject as a random effect.

Two-sided Tukey’s range tests on the mean activation or asymmetry values were then completed for the left (14 hROIs) and right (7 hROIs) hROIs to identify the origins of the significant effects and interactions found with each linear mixed-effects model.

## Results

### Task performance during the scanning session

The mean response time taken for the covert generation of the months of the year was 5261 ± 1092 ms (range 2836–7360). The mean response time taken for reading the months of the year, days and seasons was 4405 ± 603 ms (range 2703–5681). The mean response time taken for listening to the months of the year, days and seasons was 486 ± 101 ms (range 282–794). As the mean response time was calculated after the delivery of the stimulus, i.e. after 4386 ± 484 ms of Word-Lists auditory presentation, the response times in the three tasks were comparable.

### Regions showing both joint activation and joint asymmetry during production, listening and reading of Word-Lists

**Fig. 2:**
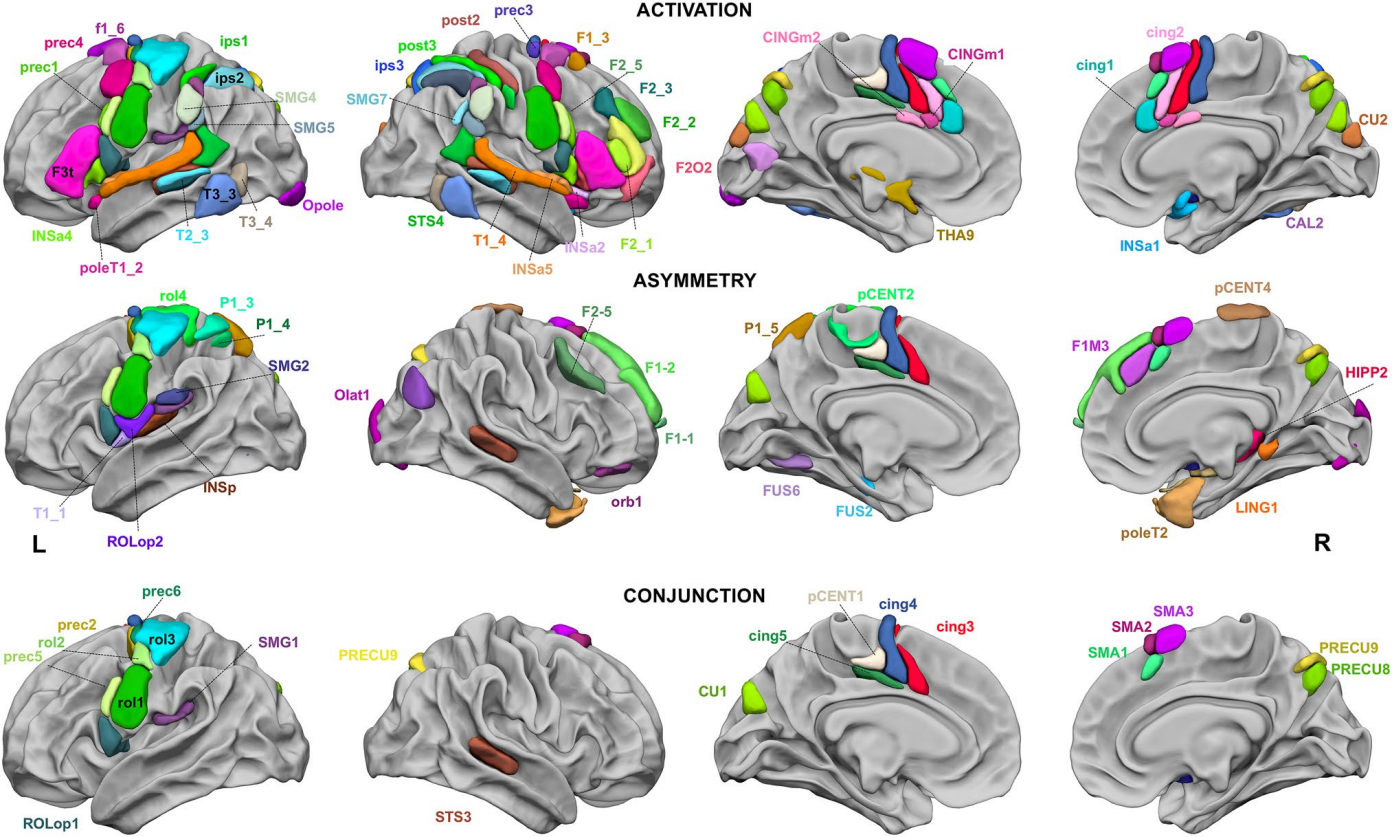
Regions of the AICHA atlas significantly activated in the three tasks (top row), significantly asymmetrical in the three tasks (middle row), and significantly conjointly activated and conjointly asymmetrical in the three tasks (bottom row). The hROIs are projected on the white matter surface of the BIL&GIN template with Surf Ice (https://www.nitrc.org/projects/surfice/) software. Leftward asymmetrical hROIs, as well as conjointly leftward activated and conjointly asymmetrical hROIs, are presented on the left hemisphere, and rightward asymmetrical hROIs are presented on the right hemisphere. The hROIs considered activated or asymmetrical per hemisphere by task were selected according to a *p* value < 0.05; the statistical threshold applied to the conjunction of asymmetry and activation for a given task was *p* < 0.0025 for each hemisphere, and the threshold set for the three-task conjunction was *p* < 6.25 × 10^−6^

The first observation was that 14 leftward hROIs showed both joint leftward activation and joint leftward asymmetry, while 7 rightward hROIs showed both joint rightward activation and joint rightward asymmetry, demonstrating the left hemisphere dominance of brain areas dedicated to Word-List processing (Fig. 2, Table 1).

**Table 1:** Number of hROIs identified at each selection step

Contrasts	L activation	L asymmetry	L activation and asymmetry
PROD	76 (94)	22 (41)	18 (26)
LISN	75 (86)	36 (62)	20 (37)
READ	89 (101)	39 (58)	31 (39)
Conjunction 3 contrasts	63	42	14

Contrasts	R activation	R asymmetry	R activation and asymmetry
PROD	85 (106)	54 (83)	39 (58)
LISN	66 (85)	26 (52)	17 (29)
READ	93 (110)	40 (68)	25 (44)
Conjunction 3 contrasts	52	21	7

a. Number of hROIs having significant left activation, leftward asymmetry or conjunction of activation and asymmetry for the three Word-List tasks. b. Number of hROIs having significant right activation, rightward asymmetry or conjunction of activation and asymmetry for the three Word-List tasks. For a given contrast or asymmetry and for a given hemisphere, the statistical threshold was set at 0.00027 (Bonferroni correction for 184 hROIs). For the conjunction between activation and asymmetry the statistical threshold was set at 0.016 (Bonferroni correction for the conjunction of two contrasts) and for the conjunction between three tasks, the statistical threshold was set at 0.05 (Bonferroni correction the conjunction of three tasks). The number of regions at a non-corrected threshold of 0.05 is given in brackets

#### Left hemisphere

The conjunction of significant leftward activation in the three contrasts (PROD ∩ LISN ∩ READ) revealed 50 left hROIs (Fig. 2, top row). The conjunction of a significant leftward asymmetry in the three contrasts revealed 26 hROIs (Fig. 2, middle row), and 14 left hROIs showed both joint leftward activation and joint leftward asymmetry (*p* < 1.56 × 10^−8^; Fig. 2 bottom row, Table 1). Most of these hROIs were located in the frontal cortex, including seven hROIs straddling the Rolandic sulcus (rol1, rol2, rol3, ROLop1) and precentral sulcus (prec2, prec5, prec6) and four located on the dorsal part of the internal surface of the frontal lobe (cing3, cing4, cing5, pCENT1). One hROI was located in the parietal lobe in the supramarginal gyrus (SMG1). Finally, one hROI was located in the occipital lobe in the cuneus (CU1). Only one subcortical area, the pallidum (PALL), was selected. Abbreviations of the hROIs names are available in Table 2. 

#### Right hemisphere

The conjunction of significant rightward activation in the three contrasts (PROD ∩ LISN ∩ READ) revealed 54 hROIs (Fig. 2, top row). The conjunction of significant rightward asymmetry revealed 19 hROIs (Fig. 2, middle row), and the conjunction of rightward activation and asymmetry revealed 7 hROIs (Fig. 2 bottom row, Table 1): 3 in the internal surface of the frontal lobe, located anteriorly to those of the left hemisphere (SMA1, SMA2, SMA3), 2 in the precuneus (PRECU8, PRECU9) and 1 in the temporal cortex (STS3) straddling the STS. Only one subcortical area, the amygdala (AMYG), was detected. Abbreviations of the hROIs names are available in Table 2.

### Activation and asymmetry profiles of the selected hROIs for the three tasks

#### Regions of the left hemisphere

There were significant main effects of Task (*p* < 0.0001), ROI (*p* < 0.0001) and Side (*p* < 0.0001), and all interactions were significant: Task × Side (*p* = 0.002), Task × ROI (*p* < 0.0001), Side × ROI (*p* < 0.0001) and Side × Task × ROI (*p* = 0.0002).

**Fig. 3:**
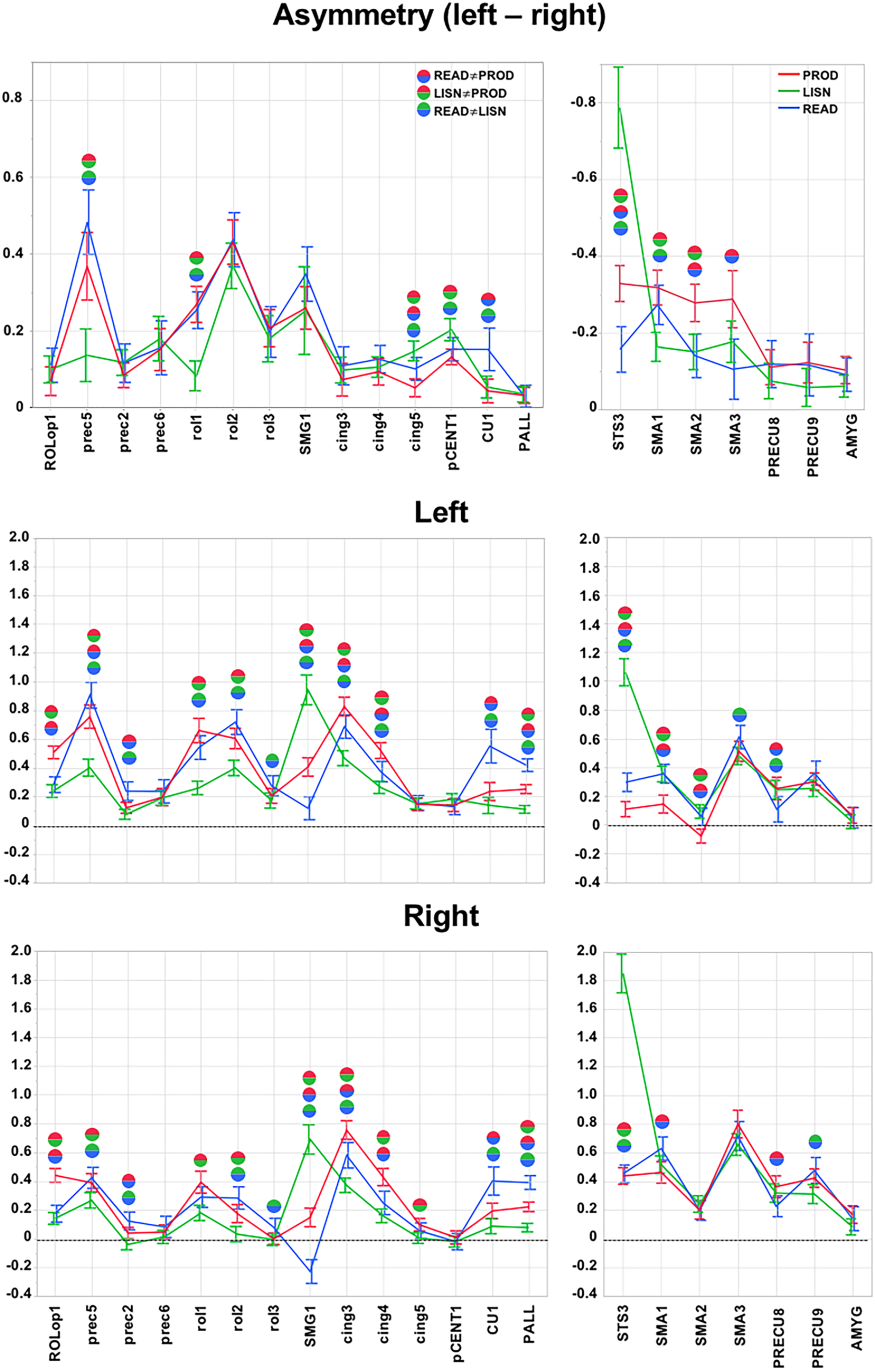
Activity and asymmetry measured in the 21 hROIs selected at step 1. The 14 hROIs selected as leftward asymmetrical and activated are displayed on the left column, and the 7 hROIs selected as rightward and asymmetrical are displayed on the right column. The top rows are the asymmetry values in each task (red corresponds to PROD, green to LISN, and blue to READ). Note that the scale of the right hROIs has been inverted to facilitate the comparison with left regions, and significant differences (Bonferroni corrected for the number of hROIs) across tasks are indicated by spheres (the blue-red sphere corresponds to a significant difference between PROD and READ, the red-green sphere corresponds to a significant difference between PROD and LISN, and the blue-green sphere corresponds to a significant difference between LISN and READ. The middle row shows the left values of activity in the left hemisphere for the same set of regions, and the bottom row shows the activity in the right hemisphere. The abbreviations correspond to those provided in Table 1

Five left regions presented modulation in asymmetry across the different modalities of the Word-List processing (Fig. 3, Table 3). The motor and premotor areas situated in the inferior part of the central sulcus, including rol1, and prec5 (located immediately anterior to rol1) were significantly more asymmetrical and activated during the tasks involving a motor component (PROD and READ) than during LISN (Fig. 3, Table 3). Two adjacent areas located in the mid-cingulate cortex, pCENT1 and cing5, presented a larger asymmetry during LISN than during READ and PROD, due to a higher right activation during PROD and/or READ than during LISN, while left activations were comparable (Fig. 3, Table 3). Finally, the anterior and dorsal part of the cuneus (CU1) was significantly more asymmetrical and more bilaterally activated during READ than during the other two tasks (Fig. 3, Table 3).

**Table 2:** Names and abbreviations of the 21 hROIs selected as either conjointly leftward activated and asymmetrical or conjointly rightward activated and asymmetrical during the three Word-List tasks

AICHA hROI name	Abbreviation	Network	*X* (mm)	*Y* (mm)	*Z* (mm)
Frontal and insula					
G_Rolandic_Oper-1-L	ROLop1	WORD_CORE	− 46.3	3.6	9.4
S_Precentral-2-L	prec2	WORD_CORE	− 25.2	− 8.5	58.9
S_Precentral-5-L	prec5	WORD_CORE	− 56.1	4.8	30.6
S_Precentral-6-L	prec6	WORD_CORE	− 29.8	− 11.2	64.7
Internal surface					
G_Cuneus-1-L	CU1	WORD_CORE	− 5.2	− 82.7	28.4
G_Paracentral_Lobule-1-L	pCENT1	WORD_CORE	− 6.9	− 16.8	50.9
G_Precuneus-8-R	PRECU8	WORD_EXE	10.5	− 68.5	41.1
G_Precuneus-9-R	PRECU9	WORD_EXE	12.7	− 68.4	50.3
G_Supp_Motor_Area-1-R	SMA1	WORD_EXE	5.9	20.5	49
G_Supp_Motor_Area-2-R	SMA2	WORD_EXE	10.5	18.5	62.9
G_Supp_Motor_Area-3-R	SMA3	WORD_EXE	6.4	10.1	66.5
S_Cingulate-3-L	cing3	WORD_CORE	− 7.4	4.5	48.5
S_Cingulate-4-L	cing4	WORD_CORE	− 7.8	− 6.3	57.4
S_Cingulate-5-L	cing5	WORD_CORE	− 8.5	− 16.4	41.6
Parietal					
G_Supramarginal-1-L	SMG1	WORD_CORE	− 54.5	− 29.5	21.4
S_Rolando-1-L	rol1	WORD_CORE	− 54.3	− 8.4	32
S_Rolando-2-L	rol2	WORD_CORE	− 43.6	− 13.7	50.6
S_Rolando-3-L	rol3	WORD_CORE	− 38.8	− 23.1	61.4
Sub-cortical					
N_Amygdala-1-R	AMYG	WORD_EXE	21.1	1.7	− 12.3
N_Pallidum-1-L	PALL	WORD_EXE	− 18.6	− 8.2	− 0.5
Temporal					
S_Sup_Temporal-3-R	STS3	WORD_CORE	53.1	− 31.9	− 0.3

The network label to which they were clustered and their coordinates in MNI space after SPM12 normalization of the AICHA atlas are also provided

A second set of motor regions showed little modulation of their asymmetry by the task modality. A first set corresponding to cing3 and cing4 showed no difference in asymmetry across tasks but higher activity during PROD and READ, which included a stronger motor component, than during LIST (Fig. 3, Table 3). A second region, ROLop1, situated in the Rolandic operculum area, presented no difference in asymmetry across tasks but showed more activity in PROD than the two other tasks (Fig. 3, Table 3). A third set, including prec2 and prec6 located immediately anterior to rol3, was also characterized by small inter-task differences in activation (Fig. 3, Table 3).

Finally, most of the regions selected in the left hemisphere exhibited higher bilateral activation during the tasks having a stronger motor component (PROD and READ) than during LISN (Fig. 3, Table 3). In addition, SMG1 bilateral activity was strongly increased by the auditory modality, with stronger activation during LISN than during the other two tasks.

#### Regions of the right hemisphere

In the right hemisphere, the main effects of Task (*p* < 0.0001), ROI (*p* < 0.0001), and Side (*p* < 0.0001) were significant, as were all interactions: Task × Side (*p* = 0.0031), Task × ROI (*p* < 0.0001), Side × ROI (*p* < 0.0001) and Side × Task × ROI (*p* < 0.0001).

Similar to the left regions, all the right hROIs showed bilateral activation except SMA2, which was deactivated on the left during PROD (Fig. 3, Table 4).

**Table 3:** Mean activation in each Word-List contrasts of the 14 hROIs showing joint left activation and left asymmetry during the three tasks (abb corresponds to the abbreviation of the AICHA hROI name provided in Table 2)

hROIs abb	PROD			LISN			READ		
	Mean (SD)	*t*	*p*	Mean (SD)	*t*	*p*	Mean (SD)	*t*	*p*
Left activation									
CU1	0.24 (0.38)	7.45	< 0.0001	0.14 (0.35)	4.87	< 0.0001	0.55 (0.71)	9.32	< 0.0001
pCENT1	0.14 (0.27)	6.36	< 0.0001	0.18 (0.24)	9.30	< 0.0001	0.13 (0.34)	4.65	< 0.0001
ROLop1	0.51 (0.27)	22.93	< 0.0001	0.24 (0.26)	10.98	< 0.0001	0.28 (0.33)	10.28	< 0.0001
SMG1	0.41 (0.39)	12.60	< 0.0001	0.94 (0.63)	17.95	< 0.0001	0.12 (0.49)	2.98	0.0034
PALL	0.25 (0.19)	16.09	< 0.0001	0.11 (0.16)	8.23	< 0.0001	0.42 (0.27)	19.00	< 0.0001
cing3	0.83 (0.39)	25.61	< 0.0001	0.47 (0.32)	17.81	< 0.0001	0.69 (0.49)	16.74	< 0.0001
cing4	0.52 (0.34)	18.71	< 0.0001	0.27 (0.26)	12.40	< 0.0001	0.38 (0.43)	10.46	< 0.0001
cing5	0.15 (0.26)	6.88	< 0.0001	0.15 (0.22)	8.32	< 0.0001	0.16 (0.31)	6.00	< 0.0001
prec2	0.12 (0.23)	6.37	< 0.0001	0.08 (0.21)	4.48	< 0.0001	0.24 (0.39)	7.30	< 0.0001
prec5	0.76 (0.49)	18.67	< 0.0001	0.40 (0.36)	13.46	< 0.0001	0.91 (0.54)	20.09	< 0.0001
prec6	0.20 (0.36)	6.54	< 0.0001	0.19 (0.32)	7.26	< 0.0001	0.24 (0.49)	5.80	< 0.0001
rol1	0.66 (0.51)	15.45	< 0.0001	0.26 (0.29)	11.01	< 0.0001	0.54 (0.50)	13.03	< 0.0001
rol2	0.61 (0.43)	16.85	< 0.0001	0.40 (0.33)	14.78	< 0.0001	0.72 (0.52)	16.55	< 0.0001
rol3	0.21 (0.31)	7.91	< 0.0001	0.17 (0.32)	6.53	< 0.0001	0.28 (0.43)	7.70	< 0.0001
Asymmetry (left–right)									
CU1	0.04 (0.19)	2.75	0.0068	0.05 (0.17)	3.70	0.0003	0.15 (0.34)	5.40	< 0.0001
pCENT1	0.13 (0.12)	12.8	< 0.0001	0.20 (0.18)	13.88	< 0.0001	0.15 (0.18)	9.89	< 0.0001
ROLop1	0.07 (0.23)	3.62	0.0004	0.10 (0.21)	5.57	< 0.0001	0.11 (0.27)	4.86	< 0.0001
SMG1	0.26 (0.34)	9.23	< 0.0001	0.25 (0.69)	4.37	< 0.0001	0.35 (0.43)	9.69	< 0.0001
PALL	0.03 (0.13)	2.98	0.0034	0.04 (0.12)	3.36	0.0010	0.03 (0.17)	2.02	0.0451
cing3	0.07 (0.26)	3.36	0.0010	0.10 (0.20)	5.72	< 0.0001	0.11 (0.30)	4.32	< 0.0001
cing4	0.09 (0.21)	5.28	< 0.0001	0.11 (0.16)	7.77	< 0.0001	0.13 (0.22)	6.97	< 0.0001
cing5	0.05 (0.14)	4.34	< 0.0001	0.15 (0.16)	11.34	< 0.0001	0.10 (0.18)	6.70	< 0.0001
prec2	0.08 (0.20)	5.14	< 0.0001	0.12 (0.20)	6.92	< 0.0001	0.12 (0.30)	4.54	< 0.0001
prec5	0.37 (0.53)	8.26	< 0.0001	0.14 (0.42)	3.93	0.0001	0.48 (0.51)	11.42	< 0.0001
prec6	0.15 (0.33)	5.45	< 0.0001	0.18 (0.35)	6.12	< 0.0001	0.16 (0.43)	4.37	< 0.0001
rol1	0.27 (0.29)	11.29	< 0.0001	0.08 (0.24)	4.20	< 0.0001	0.25 (0.29)	10.49	< 0.0001
rol2	0.43 (0.35)	14.69	< 0.0001	0.37 (0.36)	12.32	< 0.0001	0.44 (0.43)	12.25	< 0.0001
rol3	0.21 (0.29)	8.42	< 0.0001	0.18 (5.88)	0.37	< 0.0001	0.20 (0.40)	5.88	< 0.0001

One region, STS3, located in the mid-third of the STS, presented a profile very different from all the others: STS3 presented a significantly larger rightward asymmetry during LISN than during PROD and READ (Fig. 3, Table 4). STS3 was also significantly more bilaterally activated during LISN than during the two other tasks (Fig. 3, Table 4).

Three hROIs on the internal surface of the frontal lobe, labelled SMA, located at the dorsal face and anterior part of the medial frontal gyrus, also showed a modulation of their asymmetry by the modality of the Word-List tasks. SMA1 was significantly more rightward asymmetrical during PROD and READ than during LISN, whereas SMA2 and SMA3 were significantly more asymmetrical during PROD than during the two other tasks (Fig. 3, Table 4).

**Table 4:** Mean activation and asymmetry in each Word-List contrasts of the seven hROIs showing right activation and right asymmetry during the three tasks (abb corresponds to the abbreviation of the AICHA hROI name provided in Table 2)

hROIs abb	PROD			LISN			READ		
	Mean (SD)	*t*	*p*	Mean (SD)	*t*	*p*	Mean (SD)	*t*	*p*
Left activation									
PRECU8	0.36 (0.46)	9.48	< 0.0001	0.32 (0.39)	9.85	< 0.0001	0.23 (0.44)	6.18	< 0.0001
PRECU9	0.42 (0.39)	13.04	< 0.0001	0.31 (0.4)	9.34	< 0.0001	0.48 (0.55)	10.42	< 0.0001
SMA1	0.46 (0.45)	12.24	< 0.0001	0.52 (0.37)	16.83	< 0.0001	0.63 (0.49)	15.36	< 0.0001
SMA2	0.20 (0.38)	6.34	< 0.0001	0.24 (0.35)	8.35	< 0.0001	0.20 (0.42)	5.70	< 0.0001
SMA3	0.80 (0.58)	16.43	< 0.0001	0.66 (0.46)	17.26	< 0.0001	0.72 (0.61)	14.04	< 0.0001
AMYG	0.17 (0.37)	5.48	< 0.0001	0.08 (0.34)	2.97	0.0035	0.14 (0.51)	3.34	0.0011
STS3	0.49 (0.35)	15.05	< 0.0001	1.85 (0.82)	27.06	< 0.0001	0.45 (0.37)	14.70	< 0.0001
Asymmetry (left–right)									
PRECU8	− 0.11 (0.28)	− 4.77	< 0.0001	− 0.07 (0.28)	− 3.18	0.0018	− 0.12 (0.37)	− 3.82	0.0002
PRECU9	− 0.12 (0.32)	− 4.55	< 0.0001	− 0.06 (0.30)	− 2.30	0.0226	− 0.12 (0.49)	− 2.85	0.0051
SMA1	− 0.32 (0.28)	− 13.85	< 0.0001	− 0.16 (0.23)	− 8.56	< 0.0001	− 0.27 (0.31)	− 10.51	< 0.0001
SMA2	− 0.28 (0.30)	− 11.28	< 0.0001	− 0.15 (0.28)	− 6.43	< 0.0001	− 0.14 (0.35)	− 4.86	< 0.0001
SMA3	− 0.29 (0.45)	− 7.64	< 0.0001	− 0.18 (0.33)	− 6.48	< 0.0001	− 0.11 (0.48)	− 2.65	0.0089
AMYG	− 0.10 (0.22)	− 5.74	< 0.0001	− 0.06 (0.17)	− 4.22	< 0.0001	− 0.09 (0.26)	− 4.13	< 0.0001
STS3	− 0.33 (0.28)	− 13.85	< 0.0001	− 0.79 (0.64)	− 14.73	< 0.0001	− 0.16 (0.36)	− 5.22	< 0.0001

In contrast, PRECU8, PRECU9 and AMYG did not show any variation in asymmetry according to the task modality.

Apart from STS3, the profiles of activation were comparable across tasks. However, there was less left activation in SMA1, SMA2 and SMA3 during PROD (with a deactivation for SMA3) and less left activation in PRECU8 during READ. On the right, activation was inferior during READ_i_ in PRECU8 and PRECU9 than during PROD and LISN, respectively, and in SMA1 during PROD than during READ.

## Part 2: intra- and inter-hemispheric connectivity at rest

### Methods

#### Participants

A subset of 138 participants [mean age: 27.3 years (SD = 6.4 years); 68 women] also completed a resting-state fMRI (rs-fMRI) acquisition lasting 8 min. Note that this resting-state acquisition was performed on average 9 months (SD = 9.6 months) before the language task acquisition in all but five cases for which the resting-state acquisition occurred approximately 1 year after the language session (range 11.2–13.8 months).

#### Resting-state image acquisition (rs-fMRI) and processing

Spontaneous brain activity was monitored for 8 min (240 volumes) using the same imaging sequence (T2*-weighted echo-planar images) as that used during the language task. Immediately prior to rs-fMRI scanning, the participants were instructed to “keep their eyes closed, to relax, to refrain from moving, to stay awake and to let their thoughts come and go” (Mazoyer et al. 2016). After pre-processing identical to that used for task-induced fMRI acquisition, time series of white matter and cerebrospinal fluid (individual average time series of voxels that belonged to each tissue class) and temporal linear trends were removed from the rs-fMRI data series using regression analysis. Additionally, rs-fMRI data were temporally filtered using a least squares linear-phase finite impulse response filter design bandpass (0.01–0.1 Hz).

For each of the 138 participants who completed the resting-state acquisition and for each of the same 185 homotopic ROIs, an individual BOLD rs-fMRI time series was computed by averaging the BOLD fMRI time series of all voxels located within the hROI volume.

From the BOLD fMRI time series of hROIs, we computed the Pearson correlation coefficients for each hROI pair of the 21 selected hROIs in each participant. We then averaged the Fisher *z*-transformed correlations among pairs of hROIs across the 138 individuals, resulting in a matrix of intrinsic connectivity for the whole population.

#### Resting-state image analysis: characterizing networks within the 21 selected hROIs

Using the same methodology as Labache et al. (2019), we applied an agglomerative hierarchical cluster analysis method on the intrinsic connectivity matrix to identify the different networks supporting the organization across the 21 hROIs. We tested the reliability of the identified networks using a multiscale bootstrap resampling method, which provides us with an approximately unbiased (AU) *p* value representing the stability of the networks based on the average matrix of intrinsic connectivity.

Finally, we calculated the average functional intrinsic correlations between the identified networks. The significance of these correlations compared to 0 was assessed using a non-parametric sign test at a significance level of 0.05 (Bonferroni correction for the number of network pairs).

## Results

### Identification of networks based on the resting-state connectivity of the 21 hROIs showing both joint leftward activation and joint leftward asymmetry or both joint rightward activation and joint rightward asymmetry during the three Word-List tasks

**Fig. 4:**
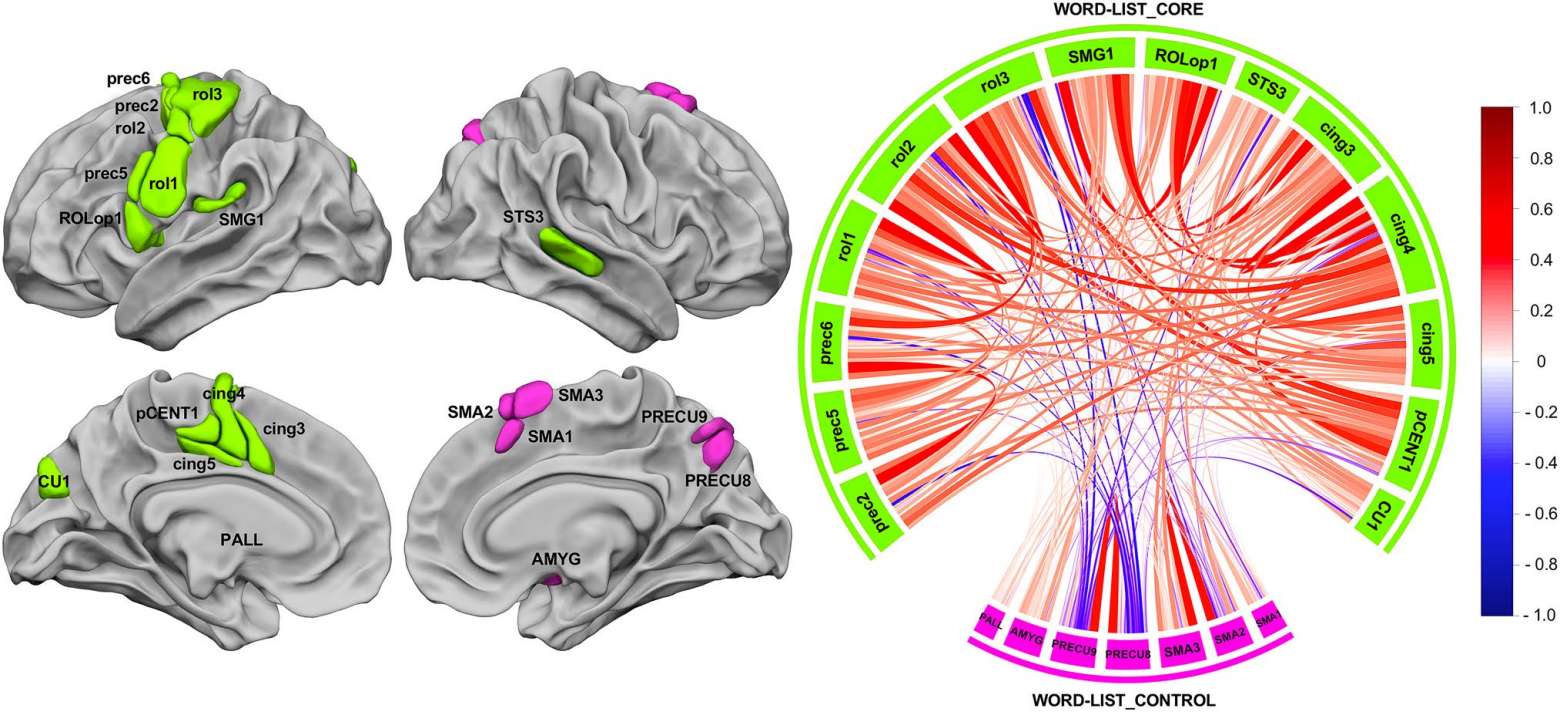
Intra- and inter-hemispheric correlations at rest across the 21 hROIs selected as either conjointly leftward activated and asymmetrical or conjointly rightward activated and asymmetrical during the three language tasks. The left motor areas and the right STS are strongly and positively correlated, and they constitute the WORD-LIST_CORE network (green) that is not significantly anticorrelated with the WORD-LIST_CONTROL network, composed of the right precuneus and SMA regions and the left pallidum (pink). The chord diagram was produced by R with the “circlize” package (Gu et al. 2014)

A hierarchical clustering analysis revealed two networks from the selected set of 21 hROIs (Fig. 4); one labelled WORD-LIST_CORE was composed of 13 left hROIs and 1 right hROI, and the other we labelled WORD-LIST_CONTROL was composed of 1 left area and 6 right hROIs.

#### The WORD-LIST_CORE network

The WORD-LIST_CORE network (Fig. 4, green) was composed of 13 hROIs hosting motor and premotor areas of the left hemisphere gathered along the Rolandic sulcus (rol1, rol2, rol3), the precentral sulcus (prec2, prec5, prec6), the Rolandic operculum (ROLop1), motor and premotor areas of the medial surface (pCENT1, cing3, cing4, cing5), the cuneus of the occipital lobe (CU1) and SMG1 of the parietal lobe. It is important to emphasize that the WORD-LIST_CORE network is also an inter-hemispheric network since it comprises the right STS3 in addition to the 13 left hROIs. We named this network WORD-LIST_CORE because it included essential phonological processing regions, as further described below. WORD-LIST_CORE was the largest network in terms of volume (53136 mm^3^), as it was 4.05 times larger than WORD-LIST_CONTROL (13104 mm^3^).

ROLop1 appeared to be a very important hROI for communication within this network since it was among the top 5% of the strongest correlations among non-contiguous hROIs (ROLop1–SMG1: *r* = 0.62, ROLop1–cing3: *r* = 0.59, ROLop1–cing4: *r* = 0.52). It is interesting to note that the SMG1–cing4 correlation (*r* = 0.53) was also among the 5% highest correlations underlying a very strong antero-posterior tripartite connection between ROLop1, SMG1 and cing4.

#### The WORD-LIST_CONTROL network

The second network consisted of seven hROIs: six right hROIs including the three superior motor areas of the internal frontal lobe surface (SMA1, SMA2 and SMA3, which is SMA proper), the posterior part of the precuneus (PRECU8 and PRECU9) and the AMYG. In addition to these right areas, the left PALL was also part of the network. We labelled this network WORD-LIST_CONTROL because it included regions involved in the monitoring aspect of the tasks that were common to the three tasks, that is to say, maintaining the instructions, detecting the end of each word-list task and clicking on the response pad.

Note that the AU *p* values provided by the multiscale bootstrap resampling method showed that both networks were 81% reliable.

### Temporal correlation across networks and significance

The chord diagram shown in Fig. 4 describes the average correlations between each pair of hROIs in the two networks. A non-significant negative mean intrinsic correlation was found between WORD-LIST_CORE and WORD-LIST_CONTROL (*R* = − 0.04; 55.07% of the participants showed a negative correlation, *p* = 0.13).

## Summary of the results of the whole study

The analysis of the connectivity at rest across the 21 hROIs common to the production, listening and reading of over-learnt lists of words made it possible to identify a set of two networks. A large WORD-LIST_CORE network made of plurimodal areas was revealed and included in the left hemisphere, premotor and motor regions that were more activated during PROD and READ than during LISN, an auditory area situated in the anterior part of the left SMG that was more activated during LISN than during PROD and READ, and a visual region, CU1 that was more activated and more asymmetrical during READ than during the two other tasks. Importantly, the strongest correlations at rest between these 21 hROIs were observed across the anterior and posterior areas (action–perception), namely, Rolop1 and cing4 with SMG1. In the right hemisphere, the WORD-LIST_CORE network included STS3, located in the mid-third of the sulcus, which was significantly more activated and asymmetrical during LISN than during PROD and READ. Note that a second network (WORD-LIST_CONTROL), composed of the right SMA1 and SMA2 (located at the preSMA), SMA3 (SMA proper) and precuneus, as well as the left PALL, was identified and was not correlated with the WORD-LIST_CORE network. The areas comprising this WORD-LIST_CONTROL network are mainly involved in extra linguistic, executive processes and attentional systems recruited to manage task completion.

## Discussion

Here, we demonstrate a large-scale network of areas commonly shared by the production, listening and reading of lists of words. This network includes not only articulatory and auditory areas, but also a region of the visual cortex, the cuneus. Though more recruited during the reading task, this region, considered to be a component of accurate phonological awareness (Bolger et al. 2008), is also involved in word-list articulation and listening. During human development, speech perception and speech production, engaging auditory and motor (articulation) modalities, are initially linked together. The subsequent acquisition of reading skills, engaging the visual modality, is built upon these two components. The present results show a brain organization in adults that is a reflection of the whole developmental and learning processes of language, where action and perception circuits are interdependent and organized in networks, among which a trace of the learning modality is still present in the brain.

We will first discuss the WORD-LIST_CORE network from the left motor and premotor areas involved in action, up to the involvement of the audio–motor loop extending to the phonological loop and the right STS.

## WORD-LIST_CORE network underpinning supramodal WORD-LIST processing

### Left motor and premotor areas: from the speech effector areas to the hand area

On the action side, the results revealed seven areas straddling the Rolandic sulcus and precentral gyrus and four others located at the internal surface of the frontal lobe, all of which were modulated by the mere nature of the task and dependent on the modality.

**Fig. 5:**
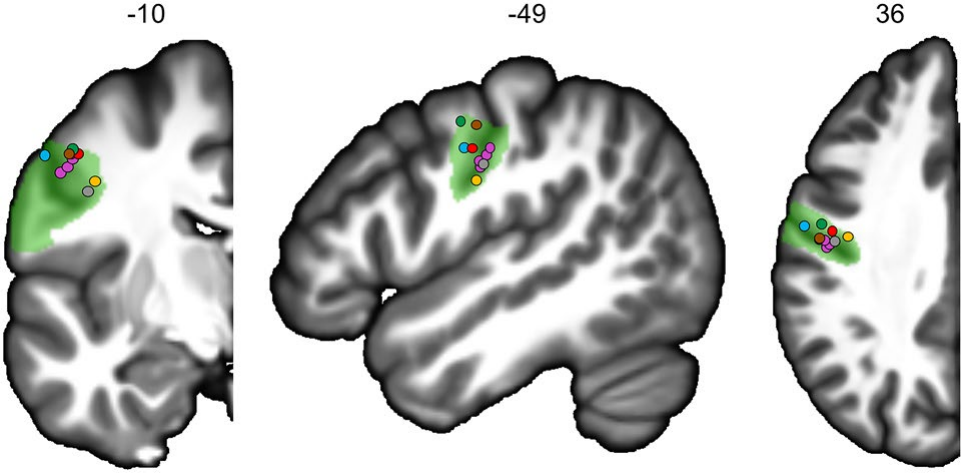
Locations of the activation peaks from five studies on the left hemisphere coronal, sagittal and axial slices from the BIL&GIN display template; the hROI numbers correspond to the *x*, *y* and *z*-axes in the MNI space. In green, the representation of rol1. Larynx areas are in blue (Brown et al. 2009) and orange (Brown et al. 2008). In red, the mouth area (Fox et al. 2001). In purple, the lips, tongue, jaw and vowels areas (Grabski et al. 2012). In brown-grey, the activation peak of the motor areas for speech production (4p and 4a/6), and in green, the activation peak of the superior part of the ventral premotor cortex during listening to speech (Wilson et al. 2004)

The regions with the strongest motor involvement were located along the Rolandic sulcus and included primary motor areas that showed very large activations during PROD and READ. This is in accordance with Penfield’s cortical stimulation studies, which provided the first functional support for the existence of somatotopy in the lower part of the Rolandic sulcus corresponding to motor control of the orofacial region (Penfield and Roberts, 1959). In fact, rol1 and adjacent rol2 match not only the area involved in speech production as described by Wilson (Wilson et al. 2004), but also the areas involved in mouth, larynx, tongue, jaw and lip movements, as reported in several studies (Brown et al. 2009, 2008; Fox et al. 2001; Grabski et al. 2012; Wilson et al. 2004) (Fig. 5). More precisely, along the dorsal-to-ventral orientation of rol1, the somatotopic representation of speech listening and mouth, lips, jaw, tongue and larynx movements clearly resembles the somatotopic organization of speech effectors proposed by Conant and collaborators (Conant et al. 2014). Moreover, the strongest asymmetry in this area during PROD and READ is consistent with the fact that these two tasks involved covert articulation or subvocalization. The premotor area prec5, tightly linked to rol1 along with cing3, was characterized by a very strong BOLD increase during READ compared to both PROD and LISN, suggesting an involvement of motor programming and articulatory coding (Dietz et al. 2005; Price 2012). Furthermore, damage to a similar region in the premotor cortex in a sample of 106 patients with diverse profiles of aphasia was shown to be strongly correlated with phonological naming errors (Schwartz et al. 2012). On the internal surface, cing3 and cing4, partly overlapping the SMA tongue area, and the anterior part of the mid-cingulate cortex situated at the tongue cingulate motor area according to Amiez et al. (2014), also presented a strong motor component, as revealed by their increased activation in PROD and READ compared to LISN. In addition, the left PALL was more activated during READ than during the two other tasks, which is in accordance with results from a meta-analysis revealing that, compared to children, adult readers recruit a larger network, including the left PALL (Paulesu et al. 2014). Importantly, the same region has been shown to be involved in the audio–motor adjustments during auditory feedback control of speech, confirming its involvement in plurimodal modulation (Tourville et al. 2008).

On the most ventral part of the Rolandic sulcus, ROLop1 presented a greater involvement in PROD than in the other tasks. This region has been reported to be activated by overt and covert articulation (Heim et al. 2002; Herbster et al. 1997; Price et al. 1996) and to be involved in phonological rehearsal (Veroude et al. 2010) and in silent recitation of the months of the year (Wildgruber et al. 1996); moreover, lesions in this region have been associated with articulatory disorders (Tonkonogy and Goodglass 1981), which is in accordance with the motor component revealed in the present study. The lower activation of ROLop1 compared to rol1 and prec5 was because this region is a secondary motor area. It could also be suggested that ROLop1 activation could implement a simulation of phoneme production according to the model by Wilson and Iacoboni (2006), which stipulated that the prediction of the acoustic consequences of phoneme production can be compared in the superior temporal cortex with the acoustic analysis of the speech sounds heard. In the present study, we cannot disentangle an actual motor component related to subvocalization from a simulation, both being potentially engaged depending on the task: simulation during the listening task and vocalization during the production task as well as a very likely vocalization aspect during the reading task. Moreover, within the WORD-LIST_CORE network, intrinsic connectivity at rest revealed that ROLop1 was an essential hROI for communication within this network, as it was particularly correlated not only with the mid-cingulate cortex tongue areas cing3 and cing4, but also with SMG1.

On the dorsal part, a last set of areas, the prec2 and prec3 premotor areas situated along the precentral sulcus and tightly linked to rol3, which is at the location of the hand-motor area (Mellet et al. 2016), presented joint activation during the three tasks and did not show strong differences across tasks. On the internal surface, pCENT1 and cing5, located in the mid-cingulate cortex, have also been shown to be involved in hand representation (Amiez and Petrides 2014). Given their functional location, one explanation concerning the language ontogenesis observations could stipulate that the motor control of the vocal tract (speech articulators) and the motor control of the hand develop in cooperation, arguing for a hand–mouth gestural basis for language (Iverson 2010). An alternative explanation would be in relation to the motor response provided by the subjects at the end of each Word-List task; however, as the central cross-change detection is likely to cancel out most of these non-specific hand-motor activations and as there is a lack of somatosensory activation, it does not seem to support the latter hypothesis.

### Involvement of the audio–motor loop and extension to the phonological loop during READ

The results of the intrinsic connectivity showed a strong correlation between SMG1 and cing4: this underlies a very strong antero-posterior tripartite connection between ROLop1, SMG1 and cing4. Therefore, there does exist a group of hROIs common to the three Word-List tasks, with a leftward involvement of brain areas specifically involved in word processing. These areas, found to be strongly and positively synchronized at rest, constitute a network including the more anterior left SMG hROI, namely, SMG1, as well as the frontal and cingulate motor and premotor areas mentioned above. These frontal and temporal regions, connected through the arcuate fasciculus tract (Catani et al. 2005), appear to consist of hROIs involved in the plurimodal representation of speech sound processing, the so-called audio–motor loop (Vigneau et al. 2006). The left areas of the audio–motor network, conjointly recruited during the three Word-List tasks, correspond at large to the neural bases of a perception–action cycle for speech sounds. Such a model, based on the link between speech perception and speech production, posits that articulatory gestures are the central motor units on which both speech perception and production develop and act according to the motor theory of speech (Liberman and Whalen 2000). More recently, neurobiological theories of speech perception have proposed a more dynamic and integrative model in which language processing relies on action–perception circuits distributed across the auditory and motor systems (Pulvermüller and Fadiga 2010, 2016). Within the WORD-LIST_CORE network, areas hosting mostly motor and premotor areas were also activated during LISN, although at a lower intensity and with a lower asymmetry. If we regard the recruitment of the SMG1 in the production task, all this taken together favours the theory supporting an involvement of action–perception circuits regardless of the Word-List task (Pulvermüller and Fadiga 2010, 2016). It is interesting to observe that READ also appears to recruit this action–perception circuit, revealing a significant activation in rol1 compared to LISN. Interestingly, this circuit, posited to subserve the mapping between sensory and motor phonological codes, is also engaged in reading (Danelli et al. 2015; Malins et al. 2016). The action–perception circuit recruited in the present study for READ also reflects the fact that the participants were instructed to engage their attention in the reading of each word and covertly articulate the lists of words at a slow speech rate. The fact that ROLop1 activation in the present study was similar for READ and LISN favours the recruitment of the action–perception circuit, and may be an indication that while reading these over-learnt words, the subjects tended to subvocalize them, which is supported by the gradient of significant activation in rol1 (production > reading > listening).

Within the large perception–action model of Fuster (2001, 2014), the literature has identified a set of areas that are considered the neural support of the phonological working memory loop postulated by Baddeley et al. (1998). In fact, experience with the Word-List automatically engages working memory processes and that component is common to the three word tasks. On the perception side, one cluster (SMG1) was more activated and asymmetrical in LISN than in READ and PROD. Interestingly, the SMG1 is situated on the posterior part of the planum temporale, corresponding in the literature to the Sylvian–parietal–temporal area or Spt (Buchsbaum et al. 2011; Yue et al. 2018), and has been described as a sensory–motor integration area for the vocal tract. Area Spt would be part of the phonological loop described by Baddeley et al. (1998), in which the content of the phonological store can be kept active via articulatory rehearsal (Buchsbaum et al. 2011). More precisely, the Spt area has been assumed to have a storage function (Martin 2005; Smith and Jonides 1998) and to play an important role in the short-term storage of phonological representations by serving as a phonological buffer (Yue et al. 2018). The activity of area Spt is thought to be correlated with some frontal speech production areas even if their precise locations differ across studies: the pars opercularis according to Buchsbaum et al. (2001) and Poldrack et al. (Poldrack et al. 1999) and the dorsal part of the pars triangularis of the inferior frontal gyrus (F3t) according to the meta-analysis by Vigneau et al. (2006). It is noteworthy that F3t was bilaterally activated during the three tasks in the present study (Fig. 2, top row); this bilateral involvement impeded conjoint activation and asymmetry. Together with the F3t activation, on the action side, prec5 was found to show both joint leftward activation and joint leftward asymmetry during the three Word-List tasks, with a gradient of activation ranging from more activation for READ and PROD and less activation for LISN. This premotor area, prec5, has been proposed to make up a subvocal rehearsal system (Chein and Fiez 2001) and/or to support executive processes allowing the maintenance of content in verbal working memory (Smith and Jonides 1998), which is in accordance with the highest levels of activation during READ and PROD in our study. Furthermore, a single rTMS intervention targeting either the left SMG or the left posterior part of the inferior frontal gyrus, which are considered phonological nodes, was sufficient to disrupt phonological decisions, providing further support for the view that both regions contribute to efficient phonological decisions, particularly subvocal articulation (Hartwigsen et al. 2016).

### Activation of the right STS3 involved in prosodic integration during PROD and READ and strong connection of the right STS3 with motor areas within the WORD-LIST_CORE network

The rightward conjoint activation of STS3 as well as its rightward asymmetry, though more activated in LISN but less activated in READ, could be accounted for by a rightward preference for non-linguistic information such as tonal prosody (Belin et al. 2002). More particularly, the right STS3, which is located in the mid-part of the STS, closely matched the activation peak showing rightward asymmetry described by Beaucousin and others (2007). This area overlaps the posterior human voice area (pHVA, Pernet et al. 2015) and corresponds to the posterior voice area described by Bodin et al. (2018). The pHVA is located on a sulcal pit corresponding to the place of the larger sulcal depth. In addition, the rightward asymmetry of this sulcal depth is specific to humans and exists regardless of the age of the subjects (infants, children or adults) (Leroy et al. 2015). The present study brings new understanding on the precise role of this region, which is not fully understood as yet. We propose that the metrics of the lists of words, resembling a reciting, are processed in this area, which is supported by its greater activation during LISN. This is in accordance with the auditory material presented to the participants, that is, the list being spoken along with the specific prosody of a list. Moreover, this region was more activated during a prosodic task than during a phonetic task (Sammler et al. 2015). During speech, words present non-verbal prosodic information that is intertwined with verbal information (Kotz and Paulmann 2007; Pell and Kotz 2011). Furthermore, prosodic and verbal cues in speech differ in their spectro-temporal properties: prosodic cues consist of slow spectral changes (over more than 200 ms) and phonemic cues consist of fast spectral changes (less than 50 ms). The right hemisphere has been suggested to be more sensitive to fine spectral details over relatively long time scales, and the left hemisphere is more sensitive to brief spectral changes (Boemio et al. 2005; Poeppel et al. 2008; Zatorre and Belin 2001).

The link between the STS3 (corresponding to pHVA), which is a prosodic integrative area, with the left SMG and rol1 instantiated in the resting-state connectivity approach reflects the intertwining between prosodic and phonemic information. The present results are supported by a recent fMRI/ERP study that revealed that activity in the left SMG together with the central sulcus area occurred earlier than in the left superior temporal cortex during phonological processing of ambiguous speech syllables, whereas attention to the spectral (prosodic) properties of the same stimuli led to activity in the right STS (Liebenthal et al. 2016). The present study demonstrated that the pHVA was part of not only the WORD-LIST_CORE network, but also showed both rightward joint activation and rightward joint asymmetry during the three Word-List tasks. These results suggest that pHVA is involved in the tonal processing of word lists that underlie speech segmentation processing for each task (PROD, LISN and READ). Moreover, the rhythmicity of the word lists processed by the right pHVA seems to be the basis of the articulatory process, which involves the left audio–motor loop, and is consistent with the recent neuroscientific literature supporting the use of musical training. Rhythmic stimulation related to the rhythm and intonation patterns of speech (prosody) has been shown to improve auditory processing, prosodic and phonemic sensitivity in dyslexic children who perform poorly on tasks of rhythmic perception and perception of musical metres (Flaugnacco et al. 2015).

### Inclusion of the right visuospatial and attentional cortical areas supporting the executive aspects of the tasks in the bilateral WORD-LIST_CONTROL network

The brain areas constituting the second network were more related to the attentional processes conjointly shared by the three tasks, which is in accordance with the anticorrelation of the WORD-LIST_CONTROL network with the WORD-LIST_CORE network (although not significant). Among these areas, SMA1, SMA2 and SMA3 overlap the location of the supplementary frontal eye fields and partially correspond to the dorsal frontal attentional network (Corbetta and Shulman 2011). The fact that these areas were more activated on the right and showed more rightward asymmetry during PROD, which was the most effortful task, is in line with a role for attentional control. The rightward asymmetry and activation of the precuneus regions, without any between-task difference, suggested that it could be related to mental imagery triggered by the scrambled version of the picture or to episodic memory encoding in reference to the list of the days and months (Cavanna and Trimble 2006).

## General conclusion and perspectives

The present study, based on the fMRI analysis of three Word-List tasks performed by 144 healthy adult right-handers combined with the analysis of intrinsic resting-state connectivity in 138 of the same participants, makes it possible to propose, for the first time, a model of the neural organization of Word-List processing during production, listening and reading tasks. This model posits that (1) action and perception circuits are interdependent and organized in networks, among which a trace of the learning modality is still present in the brain; (2) the involvement of phonological action–perception circuits, such as the phonological working memory loop, in which articulatory gestures are the central motor units on which word perception, production and reading develop and act according to the motor theory of speech (Liberman and Whalen 2000), as revealed by the recruitment of leftward frontal and precentral areas together with temporo-parietal areas, and (3) the involvement of the left SMG with the right STS3 (pHVA), which is a prosodic integrative area, could reflect the intertwining between prosodic and phonemic information. The set of regions that constitutes the Word-list Multimodal Cortical Atlas (WMCA) is available for download at http://www.gin.cnrs.fr/fr/outils/wmca/.

## Data Availability

The datasets analysed in the current study to obtain the atlas are not publicly available because they were established by the laboratory. They will be available when the laboratory completes all the publications. The atlas is publicly available at http://www.gin.cnrs.fr/en/tools/.
